# Stimulus electrodiagnosis and motor and functional evaluations during ulnar nerve recovery

**DOI:** 10.1590/bjpt-rbf.2014.0138

**Published:** 2016-01-19

**Authors:** Luciane F. R. M. Fernandes, Nuno M. L. Oliveira, Danyelle C. S. Pelet, Agnes F. S. Cunha, Marco A. S. Grecco, Luciane A. P. S. Souza

**Affiliations:** 1 Departamento de Fisioterapia Aplicada, Universidade Federal do Triângulo Mineiro (UFTM), Uberaba, MG, Brazil; 2 Curso de Fisioterapia, Universidade Federal do Triângulo Mineiro (UFTM), Uberaba, MG, Brazil; 3 Departamento de Cirurgia, Universidade Federal do Triângulo Mineiro (UFTM), Uberaba, MG, Brazil

**Keywords:** chronaxie, ulnar nerve, evaluation studies, disability evaluation, rehabilitation, movement

## Abstract

**BACKGROUND:**

Distal ulnar nerve injury leads to impairment of hand function due to motor and sensorial changes. Stimulus electrodiagnosis (SE) is a method of assessing and monitoring the development of this type of injury.

**OBJECTIVE:**

To identify the most sensitive electrodiagnostic parameters to evaluate ulnar nerve recovery and to correlate these parameters (Rheobase, Chronaxie, and Accommodation) with motor function evaluations.

**METHOD:**

A prospective cohort study of ten patients submitted to ulnar neurorrhaphy and evaluated using electrodiagnosis and motor assessment at two moments of neural recovery. A functional evaluation using the DASH questionnaire (Disability of the Arm, Shoulder, and Hand) was conducted at the end to establish the functional status of the upper limb.

**RESULTS:**

There was significant reduction only in the Chronaxie values in relation to time of injury and side (with and without lesion), as well as significant correlation of Chronaxie with the motor domain score.

**CONCLUSION:**

Chronaxie was the most sensitive SE parameter for detecting differences in neuromuscular responses during the ulnar nerve recovery process and it was the only parameter correlated with the motor assessment.

## BULLET POINTS

- Stimulus electrodiagnosis is a reliable, noninvasive method of identifying neural regeneration.
- Chronaxie was the most sensitive parameter for assessing ulnar regeneration.
- Chronaxie and motor evaluation should be used to monitor neural regeneration.

## Introduction

Injury to the ulnar nerve is one of the most common upper limb peripheral nerve lesions. Eser et al.^1^ conducted a retrospective study and found that most cases involved lesions of the ulnar nerve, with 337 cases (27%), followed by lesions of the median nerve, with 273 cases (22%). A complete and detailed evaluation of the hand is essential in order to identify a suitable treatment and achieve the best response to therapy. Rosén and Lundborg^2^ developed a model for the specific evaluation of median and ulnar nerve lesions, considering three domains: motor, sensory, and pain/discomfort. In the motor domain, evaluation of the median and ulnar nerves involves testing the strength of key hand muscles, along with dynamometry measurements of handgrip strength.

Regarding functional evaluation, the DASH (Disability of the Arm, Shoulder, and Hand) questionnaire^3^, which consists of three modules, employs a series of questions related to different tasks involving the upper limbs. This instrument was developed to measure dysfunction and physical symptoms in the upper limbs and to evaluate progress over time^3^.

Among the various means of evaluating peripheral nerve lesions, a traditional physical therapeutic resource, which has nonetheless been infrequently utilized, is stimulus electrodiagnosis (SE). It is a reliable, noninvasive method of monitoring neural conditions and recovery progress^4^^,^ 5. In addition to its use for evaluation purposes, SE is the only resource available to establish the ideal conditions of therapeutic electrostimulation, ensuring use of the most suitable electrical pulse for treatment of a specific lesion^4^^,^ 5. SE is one of the most objective means of evaluating and monitoring the evolution of a peripheral nerve lesion^4^^,^ 6 ^,^ 7, and of guiding the use of therapeutic electrostimulation^5^. In the present study, we attempted to test the efficiency and the importance of SE as a 'tool-of-the-trade' for physical therapists, particularly in ulnar nerve recovery.

There are no reports in literature concerning the use of SE in upper limb peripheral nerve lesions. No protocols have been defined for electrostimulation, and no studies have investigated the relationship between SE parameters and the results of physical therapeutic evaluation. If a good relationship is found, it could emphasize the importance of SE in the process of nerve lesion recovery and rehabilitation. Thus, the hypothesis of this study was that the SE parameters correlate with motor scores during peripheral ulnar lesion recovery. The objective of this study, therefore, was to identify the most sensitive parameters to use for the evaluation of ulnar nerve recovery and to correlate the SE parameter values with motor performance. The neuromuscular responses, which were obtained using electrodiagnosis during the recovery process after neurorrhaphy of the ulnar nerve, were evaluated, and the electrodiagnosis parameters were correlated with the results of motor assessments. Our objective was to determine whether the motor gains are linked to neural recovery, showing a possible new use for SE.

## Method

### Subjects

An observational, prospective cohort study was carried out to investigate the recovery of the ulnar nerve after neurorrhaphy. All subjects received information about the objectives and procedures of the study and signed an informed consent form, in accordance with regulation 466/12 of the Brazilian National Health Council. The study was approved by the ethics committee of Universidade Federal do Triângulo Mineiro (UFTM), Uberaba, MG, Brazil (protocol number 1663). The inclusion criteria selected patients with ulnar nerve lesions in the region of the wrist and distal forearm and who had been submitted to neurorrhaphy and then underwent physical therapy during the first three months after surgery. The exclusion criteria were refusal to participate in the study, postoperative complications (infection), failure to attend the evaluations, and absence of muscular response during the electrodiagnosis examination.

A sample size of nine patients was determined, based on the standard deviation values of 3.03 obtained for Chronaxie in a pilot project involving five patients with ulnar nerve lesions. Sample size estimation was calculated using Power and Sample v.3.0.4 software with power of 80% and α=0.05. The Chronaxie variable was selected due to its recognized importance in electrodiagnostic examinations^4^^,^ 8 ^,^ 9.

The study was conducted with ten patients, including six men and four women with a mean age of 42 (SD=15) years. In the sample, only one subject was left-handed, and the right-hand side was more severely affected by lesions acquired in the workplace. All of the patients underwent neurorrhaphy, carried out by the same medical team, and were referred to the same physical therapy service at the UFTM, which followed the same protocol developed for the study. Nine patients underwent surgery in the first three weeks following injury.

Two evaluations of the injured limb were performed: initial (EV1) and final (EV2). EV1 was conducted during the initial phase (between 4 and 6 months post-surgery) and EV2 was performed at a later stage (between 10 and 15 months post-surgery). Evaluations (denoted EVWL) were also made on the contralateral limb (i.e. without lesion). All evaluations were conducted by the same examiner and under the same conditions.

### Equipment and functional evaluation

The equipment used for the SE tests included: a) a universal pulse generator (Model Nemesys 941, Quark, Brazil); b) aluminum electrodes (10×5 cm); c) natural plant sponges (10×5 cm); d) an electrodiagnosis pen; and e) evaluation sheets (available from the instrument manual). For the motor assessment, a hydraulic dynamometer (Jamar^(r))^ was used, and the functional evaluation was conducted using the DASH questionnaire that had been translated and validated for use in Portuguese^10^^,^ 11.

### Electrodiagnostic testing

The parameters considered in the SE were Rheobase, Chronaxie, and Accommodation. Rheobase corresponds to the minimum stimulation intensity able to produce the smallest muscular contraction that can be perceived visually. This was achieved using a rectangular pulse with a period (T) of 1.0 s and an interval between pulses (R) of 2.0 s^8^^,^ 12 ^,^ 13. Chronaxie corresponds to the shortest time necessary to produce a muscular contraction, also using a rectangular pulse with an interval of 2.0 s and an amplitude equal to two times the Rheobase obtained previously^8^^,^ 12 ^,^ 14. Accommodation is defined similarly to Rheobase, but the measurement is performed using an exponential pulse with a period of 1.0 s and an interval between the pulses of 2.0 s^8^^,^ 14. Both Rheobase and Accommodation are measures of intensity and are given in units of milliamps (mA), while Chronaxie is a measure of the duration or width of the pulse and is therefore given in milliseconds (ms)^8^.

The subjects were placed in the seated position with the upper limb supported and maintaining the shoulder adduced, the elbow flexed at 90°, the forearm supine, and the wrist in a neutral position. First, the skin was cleansed with 70% alcohol in order to reduce its impedance. The muscle evaluated was the abductor of the fifth finger. An SMS (strong muscle stimulation) current was used to locate the motor point, employing a monopolar technique with two electrodes. One was a pen-type (active) electrode with an area sufficiently small to be able to stimulate the abductor muscle of the fifth finger. Dampened gauze was used to cover the metal tip in order to avoid direct contact with the skin. The other (passive) dispersive electrode had a greater area (in order to diminish the concentration of the electric charge on the skin) and was attached to the contralateral upper limb with an elastic band. The interface between this electrode and the skin was filled with a dampened sponge on the contractile part of the brachial biceps in order to close the circuit, following the recommendations provided in the manufacturer's manual. The stimulation electrode was positioned perpendicularly to the muscle under evaluation, and the pressure and angle of the pen were set after the motor point had been located. The intensity used was sufficient to induce a visible contraction. The SE was then initiated and the Rheobase, Chronaxie, and Accommodation values were recorded. The test was performed bilaterally, with the contralateral side used as the control.

### Evaluation of motor performance

Hand muscle strength and grip strength were determined according to the standardized Rosén and Lundborg motor score procedure^2^. The muscles used to evaluate hand strength were the abductor of the fifth finger, the fourth palmar, and the first dorsal interosseous. The results were graded from zero to five, according to the Highet scale^15^, and the values obtained for the three muscles were added and divided by 15 (the value for a normal individual).

The position adopted for measurements of grip strength was that recommended by the American Society of Hand Therapists (ASHT)^16^. Three measurements were performed and the arithmetic mean was calculated and divided by the mean for the healthy side.

### Functional evaluation

In this study, only the module of the DASH questionnaire that evaluates functional ability was used. The score obtained varies from 0 to 100%, and the higher the score is, the greater the functional limitation^3^^,^ 10 ^,^ 11. The DASH test was only used in the final evaluation, in order to measure and describe functional status.

### Data analysis

The normality of the SE data (Rheobase, Chronaxie, and Accommodation) was assessed using the Shapiro-Wilks test, and only the Chronaxie values were shown not to have normal distribution. Mean, standard deviation (SD), median, and maximum and minimum values were obtained through descriptive analysis. For the Chronaxie inferential analysis, the Wilcoxon matched pair (time) and Mann-Whitney U test for independent samples (side) were used. For Rheobase and Accommodation inferential analysis, the Student t test for dependent samples (time) and the Student t test for independent samples (side) were used. Finally, we calculated the correlation between the SE data and the Rosén and Lundborg^2^ motor domain scores using the Spearman rank test. For all the tests, the significance level was set at 5%. The software Statistica 7 was used for all analyses.

## Results

**Figure 1:**
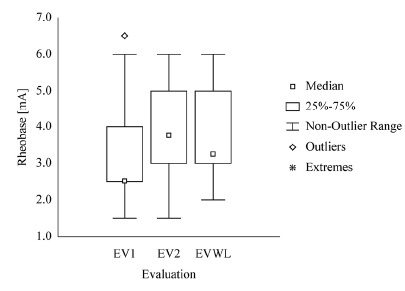
- EV1=Initial Evaluation. EV2=Final Evaluation. rs=Spearman correlation coefficient. *significant at p<0.05.

**Figure 2:**
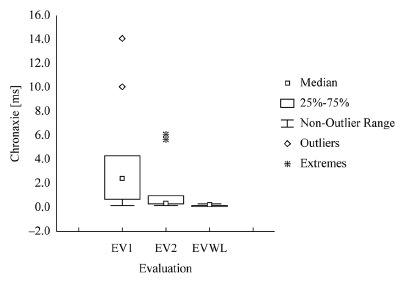
- Boxplot of values of the initial (EV1), final (EV2), and side without lesion (EVWL) evaluations for Chronaxie

**Figure 3:**
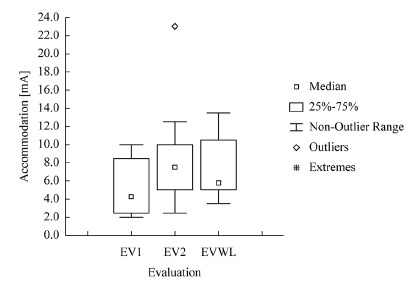
- Boxplot of values of the initial (EV1), final (EV2), and side without lesion (EVWL) evaluations for Accommodation.

The SE values obtained were compared considering the initial (EV1) and final (EV2) evaluations and evaluations of the sides with and without lesion (EVWL) (Figures 1-3). Chronaxie was the parameter that best represented recovery of the ulnar nerve (Figure 2).

The Chronaxie values obtained for the sides without lesion were very close to zero, and similar results were obtained in the final evaluation. The minimum, mean, standard deviation, median, and maximum values for Rheobase, Chronaxie, and Accommodation obtained in the initial (EV1), final (EV2), and side without lesion (EVWL) evaluations are shown in Table 1.

**Table 1:** - Minimum, Mean, Median, Standard Deviation (SD), and Maximum values of Rheobase, Chronaxie, and Accommodation in the initial (EV1), final (EV2), and side without lesion (EVWL) evaluations.

	Evaluation	Minimum	Mean	SD	Median	Maximum
Rheobase [mA]	EV1 EV2	1.50 1.50	3.40 3.75	1.69 1.37	2.5 3.75	6.50 6.00
	EVWL	2.00	3.85	1.43	3.25	6.00
Chronaxie [ms]	EV1 EV2	0.20 0.20	3.93 1.51	4.56 2.28	2.40 0.30	14.00 6.00
	EVWL	0.10	0.18	0.07	0.20	0.30
Accommodation [mA]	EV1	2.00	5.3	3.16	4.25	10.00
	EV2	2.50	8.7	5.94	7.50	23.00
	EVWL	3.50	7.4	3.49	5.75	15.50

According to statistical analysis, Rheobase did not present significant differences between the times of lesion EV1 and EV2 (p=0.56) or the side evaluated in EV1 (p=0.53) and in EV2 (p=0.88). Furthermore, the Accommodation values did not show any significant differences between the times of lesion (p=0.61) or between the sides (EV1: p=0.18 and EV2: p=0.56). On the other hand, the Chronaxie values were significantly different between EV1 and EV2 (p=0.01), as well as between the sides tested (EV1: p=0.00 and EV2: p=0.00).

The mean (SD) DASH values of the final evaluation were 33.1% (SD=21.3%) with minimum of 3.3% and maximum of 59.2%.

The relationship between the SE values and the Rosén and Lundborg^2^ motor domain scores was investigated by calculating the Spearman correlation coefficient (r_s_) (Table 2).

**Table 2:** - Correlations between the values of the electrodiagnosis parameters and motor domain scores).

		r_s_	P-value
EV1	Motor domain X Rheobase	0.313	0.38
	Motor domain X Chronaxie	–0.757	0.01*
	Motor domain X Accommodation	0.396	0.26
EV2	Motor domain X Rheobase	0.355	0.31
	Motor domain X Chronaxie	–0.794	0.01*
	Motor domain X Accommodation	0.247	0.49

EV1=Initial Evaluation. EV2=Final Evaluation. rs=Spearman correlation coefficient. *significant at p<0.05..

The Chronaxie parameter was the only parameter that showed a significant negative correlation with the Rosén and Lundborg^2^ motor domain score in both the initial and final evaluations.

## Discussion

This study contributes to the literature concerning quantitative evaluation of recovery of the ulnar nerve, using a test that has been largely ignored in clinical practice in recent years. SE is a resource that can be used to aid diagnosis, evaluate the stage of a lesion and nerve recovery, and define the parameters used in electrostimulation^5^. In addition to SE, the Rosén and Lundborg^2^ motor domain score and functional evaluation with the DASH questionnaire^3^^,^ 10 ^,^ 11 were used.

The profile of the assessed patients with nerve lesions was as follows: mostly males (60%); average age of 42 years; right-hand limb most commonly affected (60% of cases), and most common cause of lesion was workplace accident (50%). A similar patient profile was reported by Eser et al.^1^, who conducted a study using the data for 938 patients evaluated using electromyography and diagnosed with peripheral nerve lesions located in the upper limbs and the lower limbs. In that study, 71% of patients were male, the average age was 38 years, and the right-hand side was most frequently affected (55%). Most of the lesions (77%) were located in the upper limbs, and the main cause was car accidents (26.9%).

All patients were submitted to the same surgical procedure, which was performed by the same medical team. However, there were differences in clinical scenarios, including scarring processes and recovery times. Furthermore, the individuals showed differences in terms of both regeneration and sensitivity thresholds. Nonetheless, even considering these factors, Chronaxie proved to be sufficiently sensitive for detecting differences between the lesion phases.

It is important to emphasize that, although the SE method was described many years ago, it is rarely used in clinical practice despite the advantages described above and still requires further scientific investigation. It is likely that, in addition to a lack of information in the literature, its poor use could be related to difficulties encountered during application of the procedure. The test is detailed and requires an experienced physical therapist for its application and interpretation of the results. Another difficulty is related to the equipment required, because there are currently few options commercially available.

However, according to the initial hypothesis, the Chronaxie parameter correlates with motor scores during the recuperation of a peripheral ulnar lesion. Rheobase and Accommodation did not demonstrate any correlation. Chronaxie was the only parameter that showed significant differences between times of lesion and side with and without lesion. If only the Chronaxie test (based on Rheobase studies) was performed, which is relatively easy, it would be possible to understand the lesion and predict the motor behavior.

Chronaxie and Rheobase was first defined more than one hundred years^12^ ago, and since that time, various researchers have studied these parameters in cases of peripheral nerve lesion^4^^,^ 8 ^,^ 9 ^,^ 17 ^,^ 18. They found that Chronaxie, which provides a measure of the neuromuscular electrical excitation threshold, was the most sensitive parameter for use in detection of nerve lesions. In this study, Chronaxie was also found to be the most sensitive parameter for use in lesion diagnosis and assessment of recovery of the ulnar nerve. The behavior of this parameter during the recovery/regeneration process was similar for all the patients, with high values during the initial phase and low values during the recovery phase. In the latter case, the values were very close to those obtained for the side without lesion.

There have been few reports of Chronaxie values for patients with peripheral nerve lesion. Licht et al.^19^ associated Chronaxie values with the type and severity of lesion. The lesions were classified using six levels of severity, and the Chronaxie values were: 30-60 ms (neurotmesis); 20-30 ms (total axon degeneration); ~20 ms (partial axon degeneration); 10-20 ms (neuropraxy); 1-10 ms (moderate neuropraxy); and <1 ms (mild neuropraxy). The authors did not provide any information concerning the sample population. In this study, the Chronaxie values obtained in the initial evaluation correspond to normal physiology and moderate denervation. In the final evaluation, the values correspond to light denervation^19^.

Ervilha and Araújo^4^ conducted a study of Chronaxie using healthy individuals and individuals who had shown peripheral nerve lesions for more than eight months and less than two years. Seven muscles of the upper limb were evaluated and three Chronaxie value intervals were defined, depending on the severity of the lesion: the first (0.13 ms, SD=0.80 ms) was representative of normal individuals, the second (1.5-20 ms) reflected moderate peripheral lesion, and the third (>30 ms) indicated severe lesion and a poor prognosis. Although the duration of the lesion was considered and different muscles were evaluated with the aim of classifying all of the nerves of the upper limb, there were gaps remaining between the established intervals where Chronaxie values were not associated with lesion severity.

Therefore, Chronaxie was found to be a sensitive and useful parameter that could be used to evaluate the process of recovery/regeneration following ulnar nerve lesion. A reduction in Chronaxie towards normal values is indicative of reinnervation or the avoidance of further degeneration of the muscle fiber^8^. Here, the Chronaxie values obtained in EV2 were very close to the values obtained for the side without lesion, for all but two of the patients.

The Rheobase and Chronaxie parameters were studied by Lee et al.^20^ in patients suffering from encephalopathy after cerebrovascular accident. The results obtained for the paretic and non-paretic sides were compared, showing that the Rheobase and Chronaxie values were significantly higher for the paretic side. It could be inferred that reduction in muscular activity in cases of paresis or peripheral nerve lesion contributed to the need for greater stimulation in terms of both intensity and duration.

In the present study, the Rheobase values showed no similarity between patients or during the phases of lesion. High Rheobase values were found for both the side without lesion and in the final evaluation of some of the patients. The Accommodation parameter has not been the target of scientific studies in patients with peripheral nerve lesion, although studies have been conducted with animals^8^^,^ 9 ^,^ 21. Comparisons with the present study were, therefore, not possible.

Comparisons between SE parameters and clinical data for peripheral nerve lesions could not be found in literature. In the present study, Chronaxie showed a significant negative correlation with the values obtained for the Rosén and Lundborg^2^ motor domain score. The correlation was negative because, in the process of neural regeneration, the Chronaxie values tended to diminish towards 0.2 ms while the motor domain score increased towards 1.0. The other parameters did not show any correlation with the motor domain score.

In terms of clinical applications, the results of this study reinforce the need for detailed, quantitative and carefully directed evaluation of patients with peripheral nerve lesions. The findings also indicate the desirability to reinstate electrodiagnostic evaluation in clinical practice. Chronaxie, especially, is a valuable parameter that can be used in assessments of the recovery/regeneration process. The Chronaxie value is extremely useful for determination of the duration of the electrical impulse used for muscle stimulation and helps in the application of stimulations that are more comfortable^22^. The optimum duration of an impulse is equal to the Chronaxie of the muscle that it aims to stimulate^23^. Because the Chronaxie test requires the Rheobase value, we also have an indication of a possible stimulation intensity value.

A limitation of this study was that we did not construct the quadratic and triangular pulse graphics, which might have given us a better idea of the recuperation process. The construction of such graphics should be included in future studies. The use of DASH at baseline could also provide information about functional gain during nerve recuperation.

In conclusion, stimulus electrodiagnosis is a quantitative, noninvasive technique for neuromuscular evaluation and can be used to accompany recovery following neurorrhaphy of the ulnar nerve. The Chronaxie parameter proved to be most sensitive for identifying differences between initial and final evaluations of the limb on the lesion side, as well as between the sides with and without lesion. This parameter also presented correlation with the results of clinical motor domain assessment. The renewed use of electrodiagnosis should therefore be encouraged, and the technique should be included in both clinical practice and academic courses.
